# Editorial: Social human-robot interaction (sHRI) of human-care service robots

**DOI:** 10.3389/frobt.2022.1064440

**Published:** 2022-12-06

**Authors:** Minsu Jang, JongSuk Choi, Ho Seok Ahn, Chung Hyuk Park

**Affiliations:** ^1^ Intelligent Robotics Research Section, Electronics and Telecommunications Research Institute, Daejeon, South Korea; ^2^ AI∙Robotics Institute, Korea Institute of Science and Technology, Seoul, South Korea; ^3^ Department of Electrical Computer and Software Engineering, The University of Auckland, Auckland, New Zealand; ^4^ School of Engineering and Applied Science, George Washington University, Washington, DC, United States

**Keywords:** social human-robot interaction, human-care robots, social intelligence, social manipulation, machine learning

With the advent of an increasingly aging society and the rapid increase of distributed or single-parent families, there are increasing demands for service robots with social intelligence to be integrated into our everyday lives to improve human-care services for aging people, people who need special care, and people living alone. A consequence of this increasing demand is that robots need to have greater capabilities for social interaction and improved stable manipulability of humans and daily-use objects. It is reported that older adults and care-givers acknowledge the potential benefits of socially intelligent service robots (Broekens et al., 2009; Pino et al., 2015).

Most previous service robots have been designed to perform non-interactive and physical services such as cleaning, surveillance, delivery, etc. But now robots are intended to perform socially intelligent and interactive services like reception, guidance, emotional companionship, medical intervention and so on, which makes social human-robot interaction essential to help improve aspects of quality of life, as well as to improve the efficiency of human-care services (Clabaugh et al., 2019; Céspedes et al., 2021; Fraune et al., 2022; Niewiadomski et al., 2022).

This Research Topic received a number of submissions from research fields including HRI design, social intelligence, decision making, social psychology, and robotic social skills etc. on realizing and evaluating social aspects of both cognitive and physical human-robot interactions in our daily lives, and four of them have been accepted for publication.

Two articles in this Research Topic present machine learning-based approaches for decision making and generating behavioral responses to solicit improved social human-robot interactions.

Javed and Park investigate a methodology to automatically and intelligently initiating and maintaining engaged interactions between a dancing robot and an autistic child. The first phase of the interaction is to model and predict the engagement status of the child. A multimodal child engagement model is implemented with which engagement is predicted both in affective and task aspects. The second phase is to generate dancing interactions in two different roles: the music-driven leader role or the movement-driven follower role. Computer vision technologies including pose estimation and facial expression recognition are used to estimate the engagement levels; reinforcement learning is used for making decisions whether to take the leader role or the follower role; and an LSTM-based sequence-to-sequence model is utilized for automatically generating dancing movements from music.

Belo et al. presents a machine learning framework to train and test social skills using deep reinforcement learning in the simulation environment. Social robotics deep Q-network, short-named as SocialDQN, is proposed for training robots to continuously decide which actions to take based on social signals e.g., emotional states estimated from the visual features extracted from the face. To train robots using on-line reinforcement learning, a simulation environment is essential. A simulator called SimDRLSR (Belo and Romero, 2021) is utilized for training deep learning models based on SocialDQN. SimDRLSR provides human models and can be used to simulate social human-robot interactions, which is special as most of the previous simulators in robotics were used to simulate robot perception and control in manipulation and navigation (Nguyen and La, 2019). We find that this work suggests a novel way of implementing social interaction technologies through machine learning and simulation environments.

An article by Jeon et al. presents a method to implement a hierarchical control architecture, called combined task and motion planning (CTAMP), for enabling socially-intelligent physical manipulation by leveraging interactions between two levels of control: metric level and symbolic level. The method combines a symbolic task planner in which a sequence of action symbols is generated and a motion planner with which each action is verified based on geometric reasoning. An important capability needed to implement socially-intelligent manipulation is to make robots readily adaptable in uncertain situations. Several evaluations were conducted in three different simulation environments and the proposed method was found to be effective in generating and executing proper sequences of actions under various uncertainties and errors in robot perception and control.

The final article by Velentza et al. investigates users’ preferences on different robot personalities, interaction modalities and levels of familiarities through video stimulations-based user studies. Robot personalities include “serious type” and “cheerful type”. Interaction modalities include “expressive body movements” and “extremely friendly storytelling”. Two levels of familiarities are manipulated by first presenting participants with a robot for a short introductory session and then giving them a chance to choose whether to continue to interact with the same robot in the follow-up sessions or to change their robot partner to a new one. The responses were evaluated *via* subjective surveys and objective task performance metrics.

These four articles in this Research Topic well represent large diversity of studies in the field of social human-robot interaction for human-care robots, but the goal of the studies consistently points to the realization of human-friendly interactions for the benefit of the users. We hope these articles will provide stimulations and inspirations for further research works in the field of social human-robot interaction which would be one of the pinnacles for the future human-robot symbiosis.
